# Merkel Cell Polyomavirus (MCPyV) in the Context of Immunosuppression: Genetic Analysis of Noncoding Control Region (NCCR) Variability among a HIV-1-Positive Population

**DOI:** 10.3390/v12050507

**Published:** 2020-05-04

**Authors:** Carla Prezioso, Francisco Obregon, Donatella Ambroselli, Sara Petrolo, Paola Checconi, Donatella Maria Rodio, Luigi Coppola, Angelo Nardi, Corrado de Vito, Loredana Sarmati, Massimo Andreoni, Anna Teresa Palamara, Marco Ciotti, Valeria Pietropaolo

**Affiliations:** 1 IRCSS San Raffaele Pisana, Microbiology of Chronic Neuro-Degenerative Pathologies, 00166 Rome, Italy; preziosocarla@gmail.com (C.P.); annateresa.palamara@uniroma1.it (A.T.P.); 2 Department of Public Health and Infectious Diseases, “Sapienza” University, 00185 Rome, Italy; tavo1280@hotmail.com (F.O.); ambroselli.donatella@gmail.com (D.A.); sara_petrolo@yahoo.it (S.P.); dona.rodio@gmail.com (D.M.R.); angelo.nardi@uniroma1.it (A.N.); corrado.devito@uniroma1.it (C.d.V.); 3 IRCCS San Raffaele Pisana, Department of Human Sciences and Promotion of the Quality of Life, San Raffaele Roma Open University, 00166 Rome, Italy; paola.checconi@uniroma5.it; 4 Infectious Diseases Clinic, Policlinic Tor Vergata, 00133 Rome, Italy; luigi.coppola@ptvonline.it (L.C.); sarmati@med.uniroma2.it (L.S.); andreoni@med.uniroma2.it (M.A.); 5 Department of System Medicine, Tor Vergata University of Rome, 00133 Rome, Italy; 6 Department of Public Health and Infectious Diseases, Institute Pasteur, Cenci-Bolognetti Foundation, Sapienza University of Rome, 00185 Rome, Italy; 7 Laboratory of Clinical Microbiology and Virology, Polyclinic Tor Vergata Foundation, 00133 Rome, Italy; marco.ciotti@ptvonline.it

**Keywords:** Merkel cell polyomavirus, HIV-1-positive population, noncoding control region, GTT and GTTGA insertions, putative binding sites

## Abstract

Background: Since limited data are available about the prevalence of Merkel cell polyomavirus (MCPyV) and the genetic variability of its noncoding control region (NCCR) in the context of immunosuppression, this study aimed to investigate the distribution of MCPyV in anatomical sites other than the skin and the behavior of NCCR among an HIV-1-positive population. Methods: Urine, plasma, and rectal swabs specimens from a cohort of 66 HIV-1-positive patients were collected and subjected to quantitative real-time polymerase chain reaction (qPCR) for MCPyV DNA detection. MCPyV-positive samples were amplified by nested PCR targeting the NCCR, and NCCRs alignment was carried out to evaluate the occurrence of mutations and to identify putative binding sites for cellular factors. Results: MCPyV DNA was detected in 10/66 urine, in 7/66 plasma, and in 23/66 rectal samples, with a median value of 5 × 10^2^ copies/mL, 1.5 × 10^2^ copies/mL, and 2.3 × 10^3^ copies/mL, respectively. NCCR sequence analysis revealed a high degree of homology with the MCC350 reference strain in urine, whereas transitions, transversions, and single or double deletions were observed in plasma and rectal swabs. In these latter samples, representative GTT and GTTGA insertions were also observed. Search for putative binding sites of cellular transcription factors showed that in several strains, deletions, insertions, or single base substitutions altered the NCCR canonical configuration. Conclusions: Sequencing analysis revealed the presence of numerous mutations in the NCCR, including insertions and deletions. Whether these mutations may have an impact on the pathogenic features of the virus remains to be determined. qPCR measured on average a low viral load in the specimens analyzed, with the exception of those with the GTTGA insertion.

## 1. Introduction 

Human polyomaviruses (HPyVs) include ubiquitous, clinically silent viral pathogens that establish a symbiotic relationship with their human hosts [1]. Merkel cell polyomavirus (MCPyV) is a small, nonenveloped, double-stranded DNA virus, identified in 2008 [2] and related to the progenitors BK polyomavirus (BKPyV) and JC polyomavirus (JCPyV). Most MCPyV infections are asymptomatic, and serological studies showed that 50–80% of the healthy individuals present MCPyV-specific antibody responses [3,4]. After primary infection, HPyVs establish a lifelong persistence in different anatomical sites, such as lymphoid tissue (JCPyV), renal epithelium (JCPyV and BKPyV), and skin (MCPyV) [5]. Recently, a high prevalence of MCPyV has also been found in respiratory and stool samples from immunocompromised patients, adding important information on MCPyV prevalence and persistence in the respiratory and gastrointestinal tract [4,5,6,7,8]. MCPyV shares the genomic structure of BKPyV and JCPyV, with a circular genome divided into three functional regions: early, late, and interposed between these regions, the noncoding control region (NCCR). The early region encodes the large T antigen (LT), small T antigen (sT), 57 kT antigen (57 kT), and ALTO (alternate frame of the large T open reading frame). The late region is transcribed in the opposite direction and encodes the structural components of the virus capsid: virus protein 1 (VP1) and the minor capsid proteins virus protein 2 (VP2). MCPyV does not seem to express virus protein 3 (VP3), and virions do not seem to contain VP3, despite an in-frame internal adenin-timin-guanine (ATG) start codon in the VP2 gene [9]. The NCCR contains the origin of replication and bidirectional promoter elements [4,10,11]. The two main spliced products, LT and sT antigens, are involved in the viral infectious cycle, in cell cycle progression, and in malignant transformation of the host cell [12]. The continued expression of the MCPyV viral oncogenes sT and LT with a C-terminal truncation is required for Merkel cell carcinoma (MCC) development [13]. Although these findings provide strong evidence that MCPyV is a major causative agent of this skin cancer, whether the NCCR can influence the outcome of the infection remains elusive. It is well documented that one of the remarkable features of NCCRs is the occurrence of rearrangements, which allow to classify BKPyV and JCPyV in archetype (*ww*) and rearranged (*rr*) [14,15,16,17]. The archetype form is predominant in the urine of healthy individuals and represents the transmissible form of the virus circulating in the human population and the persistent form in the host. The *rr*-sequences, characterized by deletions and/or duplications compared with the archetype, are thought to derive from the archetype form during or after reactivation from persistence in vivo, often associated with disease [16,17]. Mutations and *rr*-forms also emerge in permissive cells in vitro [14,18,19,20,21], when replication occurs without the constraints of an effective host cellular immune response. The study of the NCCR during host immunosuppression, a critical cofactor for BKPyV, JCPyV, and MCPyV pathogenesis, is important because of the association of the *rr*-variants with specific human diseases such as polyomavirus nephropathy (PVAN) associated to BKPyV reactivation or progressive multifocal leukoencephalopathy (PML) caused by JCPyV [22,23,24]. Limited data are currently available about the possible relationship between MCPyV NCCRs mutations/rearrangements and the development of MCPyV-related disease. Hashida and coworkers evaluated the genetic variability of MCPyV NCCR in skin swab specimens of healthy individuals with distinct ethnicities and geographic origins, showing that insertions and deletions could be used to classify NCCR into five genotypes. Based on this classification, a tandem repeat was found exclusively in the NCCR of Japanese patients with MCC, while white patients from Europe or North America presented other genotypes [13]. Delbue and colleagues performed the MCPyV NCCR molecular characterization on cerebrospinal fluid (CSF) samples collected from patients affected by neurological disorders. The results obtained showed the presence of MCPyV NCCR IIc strain, according to Hashida’s NCCR classification [10]. Thus, given this background, the objective of the present study was to investigate the following: first, the prevalence of MCPyV in urine, plasma, and rectal swabs specimens among a HIV-1-positive population; and second, since little is known about NCCR alterations in MCPyV strains circulating in this population, the features of the MCPyV NCCR, focusing on NCCR variability. A search for putative binding sites of cellular transcription factors was also performed in order to verify whether mutations and/or rearrangements detected within the NCCR could fall in these binding sites. The assessment of NCCR region in different anatomical sites could help in advancing the understanding of MCPyV biology and the role of NCCR variability in the context of immunosuppression.

## 2. Materials and Methods

### 2.1. Study Population

A cross-sectional study including a cohort of 66 HIV-1-positive patients, admitted to the Infectious Diseases Clinic of the Polyclinic Tor Vergata Foundation from January 2019 to December 2019, was performed. Among the enrolled patients, 22 were new diagnoses naive to treatment and 44 were experienced patients on treatment with a triple-based antiretroviral regimen including protease/reverse transcriptase/integrase inhibitors. From this cohort (55 males/11 females, age ranged from 21 to 76 years old: mean age ± standard deviation: 40.5 years old; median: 39.9 years old), a sample of urine, plasma, and rectal swab were collected. In detail, 66 plasma, 66 urine, and 66 rectal swabs were obtained for a total of 198 specimens. Demographic and clinical characteristics are presented in Table 1. The study was approved by the local Ethic Committee of the University Hospital Tor Vergata (Rome, Italy) (protocol number 0027234/2018, 19 December 2018), and informed consent was obtained from patients.

### 2.2. MCPyV DNA Extraction and Quantitative Real-Time Polymerase Chain Reaction (qPCR) 

Total DNA was extracted from urine and plasma by DNeasy^®^ Blood & Tissue Kit (QIAGEN, S.p.A, Milan, Italy) and from rectal swab, using the Stool DNA Isolation Kit (NORGEN BIOTEK, Thorold, ON, Canada) according to the manufacturer’s instructions. The extracted nucleic acids were eluted in a final volume of 100 μL, and DNA was evaluated for its PCR suitability by amplifying the β-globin gene sequences [25]. Specific quantitative qPCR assays were performed using TaqMan-based qPCR, employing primers and probes for MCPyV sT, as previously described [26,27]. All samples were tested in triplicate, and the number of viral copies was calculated from standard curves constructed using a ten-fold dilution series of plasmids pMCV-R17a containing the entire genome of MCPyV (Addgene, #24729) (dilution range: 10^8^–10 copies/mL). The lower detection limit of the assay was 10 DNA copies of the target gene per amplification reaction, corresponding to 10 copies per reaction (10 copies/reaction). The results were reported as copies/mL.

### 2.3. MCPyV Nested PCR 

MCPyV-positive DNA samples were subjected to nested PCR for the amplification of NCCR region. Two sets of primers, ORIF1/ORIR1 (nucleotide positions 4832–4853 and nucleotide positions 5334–5314) and ORIF2/ORIR2 (nucleotide positions 5077–5100 and nucleotide positions 5280–5261), were employed to generate an NCCR fragment of 504 and 203 base pair (bp). Numbering of nucleotides was based on the sequence of MCC350, a strain of North American origin (GenBank: EU375803) [2]. In detail, PCR reactions were carried out following a published protocol [13]. PCR products were analyzed on 2% agarose gels by ethidium bromide staining. The positive PCR products were purified using the MinElute PCR Purification Kit (QIAGEN, Italy) and confirmed by sequencing, using sense and antisense primers, by a dedicated facility (Bio-Fab research s.r.l., Rome, Italy). Sequences were generated using the Big Dye Terminator Sequencing method (Life Technologies) on the ABI 3730 sequencer (Life Technologies, Monza, Italy), and analyzed with the Sequencing Analysis 5.2 software (Life Technologies).

### 2.4. NCCR Alignment and Analysis of Putative Binding Sites

The obtained sequences were compared to the reference strain (EU375803). Sequence alignment was performed using ClustalW2 [28] available on the European Molecular Biology Laboratory–European Bioinformatics Institute (EMBL-EBI) website using default parameters. The identification of putative binding sites for several transcription factors was carried out using TFBIND, available at http://tfbind.hgc.jp/ [29].

### 2.5. Statistical Analysis

MCPyV detection was summarized by counts and proportions. If continuous variables were normally distributed, they were expressed as mean ± SD; if not, they were expressed by median and range. The χ^2^ test was performed to evaluate differences for categorical variables. Group differences for continuous variables were tested using Student’s t-test or Mann–Whitney U-test, for normally and non-normally distributed variables, respectively. Associations between two continuous variables were examined by Pearson or Spearman correlation for normal or non-normal variables. Differences in viral loads, among the three anatomical sites, were analyzed using Kruskal–Wallis test. A *p*-value less than 0.05 was considered statistically significant.

## 3. Results

### 3.1. MCPyV DNA Detection and Quantification by Real-Time qPCR Analysis

Each DNA sample (66 urine, 66 plasma, and 66 rectal swabs for a total of 198 specimens) was tested for the presence of MCPyV. Overall, MCPyV DNA was detected in 10/66 (15%) urine, in 7/66 (11%) plasma, and in 23/66 (35%) rectal samples. Specifically, among the enrolled population (22 naive and 44 experienced patients), MCPyV was detected in 12 out of 22 naive and in 19 out of 44 experienced patients. In naive patients, MCPyV DNA was detected in 5/12 urine, 4/12 plasma, and in 9/12 rectal swabs. Instead, in experienced patients, MCPyV was detected in 5/19 urine, 3/19 plasma, and 14/19 rectal samples, respectively (Table 2). Overall, MCPyV DNA was found in a higher percentage among naïve versus experienced patients, either in the specific compartment or in all compartments together (urine: naïve 22.73%, experienced 11.36%; plasma: naïve 18.18%, experienced 6.82%; rectal swabs: naïve 40.91%, experienced 31.82%; overall sites: naïve 54.55%, experienced 43.18%) (Table 2). Results of qPCR showed that the median cycle threshold value (Ct) was 34.36 in urine (interquartile range (IQR) 30.08–36.07), 34.92 in plasma (IQR 33.57–36.78), whereas, in rectal swabs, median Ct was 31.57 (IQR 30.34–34.92). Analysis of qPCR results showed low amounts of viral MCPyV DNA with a median value of 5.15 × 10^2^ copies/mL (IQR 4.5 × 10^2^–5.4 × 10^2^) in urine, 1.5 × 10^2^ copies/mL (IQR 1.3 × 10^2^–2.0 × 10^2^) in plasma, and 2.3 × 10^3^ copies/mL (IQR 1.8 × 10^3^–4.1 × 10^3^) in rectal samples, with a statistical significant difference (*p* < 0.001). No significant association was found between the presence of MCPyV DNA and MC viral load versus age, gender, and HIV patient’s status (naïve/experienced) at enrollment time, although the mean age of patients with MCPyV DNA in plasma was lower than that found in patients without MCPyV DNA in plasma (*p* = 0.042). Finally, detection of MCPyV did not show a correlation with HIV-1 load at enrollment or CD4+ cell counts.

### 3.2. Genetic Analysis of NCCR 

**Figure 1:**
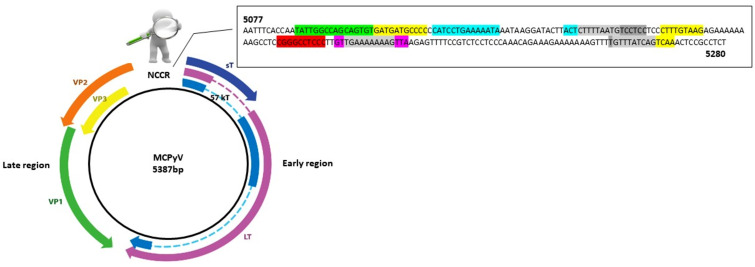
Schematic representation of the MCPyV genome (strain MCC350). The double-stranded DNA genome is 5387 bp in length. The early region encodes the large T antigen (LT), small T antigen (sT), 57 kT antigen (57 kT) and ALTO (alternate frame of the large T open reading frame). These genes function to allow for viral DNA replication. The late region is transcribed in the opposite direction and encodes the structural components of the virus capsid: virus protein 1 (VP1) and the minor capsid proteins virus protein 2 (VP2). MCPyV does not seem to express virus protein 3 (VP3), and virions do not seem to contain VP3, despite an in-frame internal ATG start codon in the VP2 gene. At the top of the figure, the amplified noncoding control region (NCCR) of 203 base pair (bp) (5077–5280 bp). Putative binding sites for cellular transcription factors are highlighted in different colors as follows: 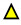
AP-1: 5104–5114 nucleotide position (5′-GATGATGCCCC-3′); 5164–5172 nucleotide position (5′-CTTTGTAAG-3′); 5257–5265 nucleotide position (5′-GTTTATCAG-3′); 5261–5269 nucleotide position (5′-ATCAGTCAA-3′); 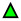
NF1: 5088–5105 nucleotide position (5′-TATTGGCCAGCAGTGTG-3′); 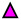
TST1: 5200–5214 nucleotide position (5′-GTTGAAAAAAAGTTA-3′); 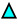
OCT1: 5116–5129 nucleotide position (5′-CATCCTGAAAAATA-3′); 5143–5146 nucleotide position (5′-ACTCTTTTAATG-3′); 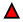
NFκB: 5188–5197 nucleotide position (5′-CGGGCCTCCC-3′); 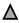
TATA-like sequences: 5146–5160 nucleotide position (5′-CTTTTAATGTCCTCC-3′); 5202–5212 nucleotide position (5′-TGAAAAAAAG-3′); 5256–5265 nucleotide position (5′-TGTTTATCAG-3′).

Analysis of MCPyV NCCR regions obtained from positive urine, plasma, and rectal swabs of HIV-1-positive subjects was carried out. The amplified NCCRs, spanning from nucleotide position 5077 to 5280, were compared with the reference sequence of the prototype North American strain MCC350 [2] (Figure 1). 

The area highlighted in light grey represents overlapping areas within one or more binding sites. Numbering of nucleotides was based on the sequence MCC350, a strain of North American origin (GenBank: EU375803) [2].

**Figure 2:**
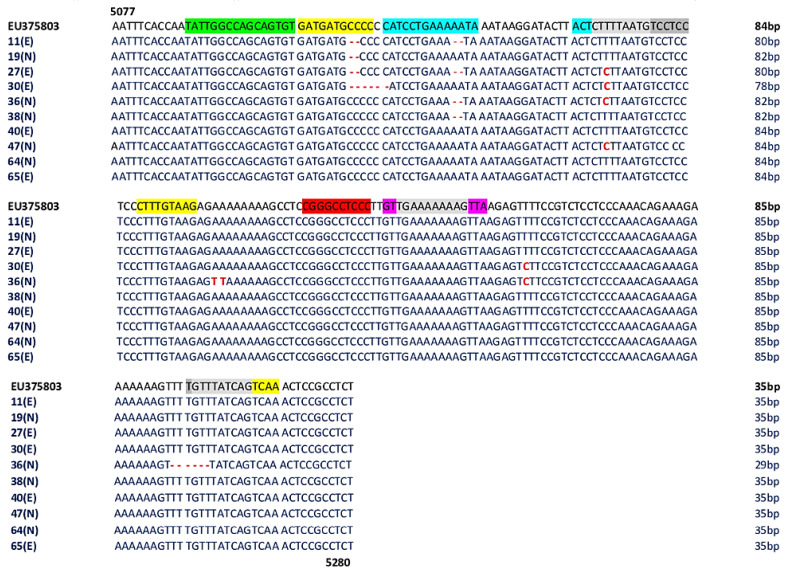
Sequence analysis of the MCPyV NCCR recovered from urine. The alignment is shown between the nucleotide sequence from 5077 to 5280 of the published sequence of MCPyV in GenBank (NCBI) (EU375803) [2] and that obtained from the sequencing of urine positive for MCPyV NCCR (*MCPyV 11,19,27,30,36,38,40,47,64,65*). 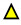
AP-1: 5104–5114 nucleotide position (5′-GATGATGCCCC-3′); 5164–5172 nucleotide position (5′-CTTTGTAAG-3′); 5257–5265 nucleotide position (5′-GTTTATCAG-3′); 5261–5269 nucleotide position (5′-ATCAGTCAA-3′); 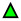
NF1: 5088–5105 nucleotide position (5′-TATTGGCCAGCAGTGTG-3′); 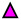
TST1: 5200–5214 nucleotide position (5′-GTTGAAAAAAAGTTA-3′); 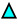
OCT1: 5116–5129 nucleotide position (5′-CATCCTGAAAAATA-3′); 5143–5146 nucleotide position (5′-ACTCTTTTAATG-3′); 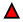
NFκB: 5188–5197 nucleotide position (5′-CGGGCCTCCC-3′); 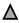
TATA-like sequences: 5146–5160 nucleotide position (5′-CTTTTAATGTCCTCC-3′); 5202–5212 nucleotide position (5′-TGAAAAAAAG-3′); 5256–5265 nucleotide position (5′-TGTTTATCAG-3′); E: experienced HIV-1-positive patients; N: naive HIV-1-positive patients.

The genetic analysis of NCCRs obtained from 10 out of 66 (15%) positive urine (*MCPyV 11,27,30,40,65* belonging to experienced (E) patients and *MCPyV 19,36,38,47,64* belonging to naive (N) patients) showed an NCCR characterized by a high degree of homology with the prototype strain, despite the presence of some deletions or mutations (Figure 2). Specifically, a 6 bp deletion (CCCCCC, positions 5111–16) in *MCPyV 30*, a 2 bp deletion (CC, positions 5111–12) in *MCPyV 11* and *19*, and a 2 bp deletion (AA, positions 5126–27) in *MCPyV 36* and *38* were observed (Figure 2). Transitions were found from nucleotide positions 5145 to 5160. In particular, 5148 T to C transition was detected in *MCPyV 27, 30, 36, 47*, 5220 T to C transition was observed in *MCPyV 30* and *36*, instead, the 5175 A to T and the 5176 A to T transversions were found in *MCPyV 36* (Figure 2)*. MCPyV 36* presented also a 6 bp (TTTTGT) deletion between nucleotides 5254–5259 (Figure 2). 

Nucleotide substitutions are in bold red text. Deletions are indicated by bold and red dashes. The highlighted in light gray represents overlapping areas within one or more binding sites. The number of base pair (bp) of each sequence is reported on the right of the Figure.

**Figure 3:**
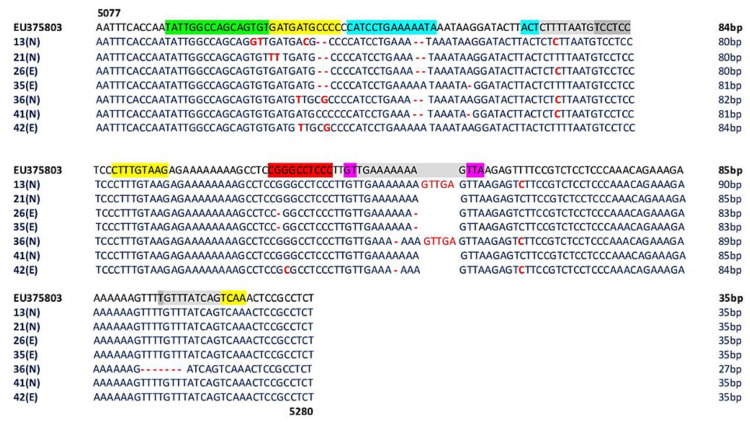
Sequence analysis of the MCPyV NCCR recovered from plasma. The alignment is shown between the nucleotide sequence from 5077 to 5280 of the published sequence of MCPyV in GenBank (NCBI) (EU375803) [2] and that obtained from the sequencing of plasma positive for MCPyV NCCR (*MCPyV 13,21,26,35,36,41,42*). 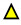
AP-1: 5104–5114 nucleotide position (5′-GATGATGCCCC-3′); 5164–5172 nucleotide position (5′-CTTTGTAAG-3′); 5257–5265 nucleotide position (5′-GTTTATCAG-3′); 5261–5269 nucleotide position (5′-ATCAGTCAA-3′); 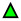
NF1: 5088–5105 nucleotide position (5′-TATTGGCCAGCAGTGTG-3′); 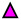
TST1: 5200–5214 nucleotide position (5′-GTTGAAAAAAAGTTA-3′); 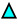
OCT1: 5116–5129 nucleotide position (5′-CATCCTGAAAAATA-3′); 5143–5146 nucleotide position (5′-ACTCTTTTAATG-3′); 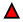
NFκB: 5188–5197 nucleotide position (5′-CGGGCCTCCC-3′); 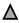
TATA-like sequences: 5146–5160 nucleotide position (5′-CTTTTAATGTCCTCC-3′); 5202–5212 nucleotide position (5′-TGAAAAAAAG-3′); 5256–5265 nucleotide position (5′-TGTTTATCAG-3′); E: experienced HIV-1-positive patients; N: naive HIV-1-positive patients.

In the positive plasma samples (7/66, 11%) (*MCPyV 26,35,42* belonging to experienced (E) patients and *MCPyV 13,21,36,41* belonging to naïve (N) patients), several mutations/deletions were found (Figure 3). In detail, the 5101 T to G transversion, the 5102 G to T transversion, the 5109 T to C transition, the 5148 T to C transition, and the 5220 T to C transition were found in *MCPyV* 13 (Figure 3). Additionally, a deletion of a 2 bp sequence (CC) at nucleotide positions 5111–5112 and a deletion of a 2 bp sequence (AA) at nucleotide positions 5126–5127 were found. Similarly, *MCPyV 21, 26* and *35* showed 2 bp deletions (CC) at nucleotide positions 5111–5112 and 5126–27 (AA). This latter deletion was also observed in strains *36* and *41* (Figure 3). Single point mutations were also observed in several strains: a 5104 G to T transversion and a 5105 A to T transversion in *MCPyV 21*, a 5108 A to T transversion in *MCPyV 36* and *42,* and a 5109 T to C transition in *MCPyV 13*. Moreover, *MCPyV 36* and *42* presented a 5112 C to G transversion, whereas the 5148 T to C transition was found in *MCPyV 13, 26, 36,* and *41*. A single 5134 A deletion was present in *MCPyV 35* and *41* (Figure 3). Additionally, the 5189 G deletion and the 5210 A deletion were detected in *MCPyV 26* and *35,* and the 5207 A deletion was found in *MCPyV 36* and *42*. The 5190 G to C transversion was found in *MCPyV 42,* and the 5220 T to C transition was found, in parallel, in *MCPyV 13, 36,* and *42* (Figure 3). Between positions 5253 and 5260, homology analysis and multiple alignments did not show differences compared with the prototype strain with the exception of *MCPyV 36* that presented an 8 bp deletion (TTTTGTTT) (Figure 3). Interestingly, a GTTGA insertion into nucleotide positions 5210–5211 was observed in *MCPyV 13* and *36*. 

Nucleotide substitutions are in bold red text. Deletions are indicated by bold and red dashes The area highlighted in light gray represents overlapping areas within one or more binding sites. The number of base pair (bp) of each sequence is reported on the right of the Figure.

**Figure 4:**
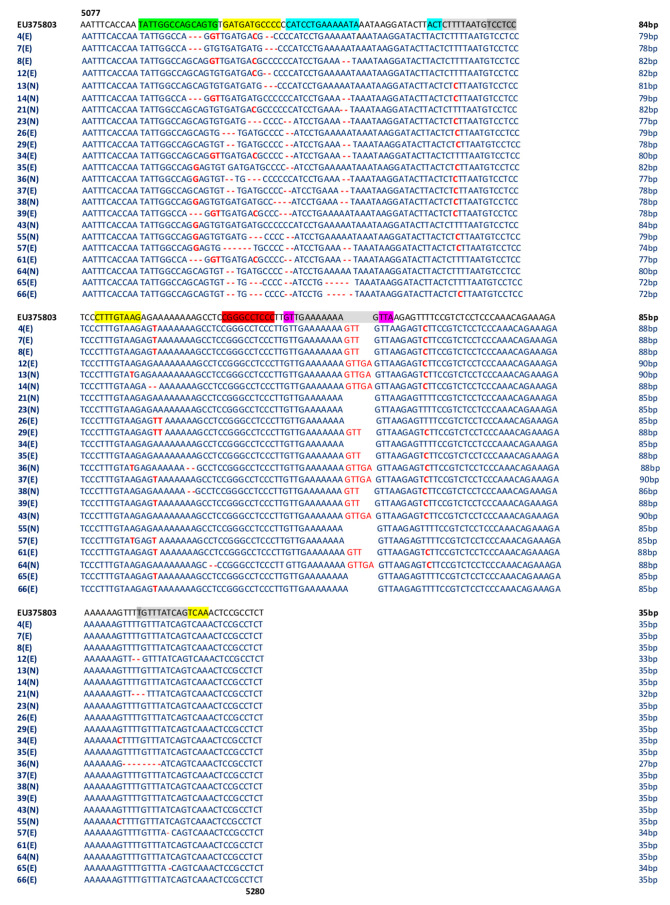
Sequence analysis of the MCPyV NCCR recovered from rectal swabs. The alignment is shown between the nucleotide sequence from 5077 to 5280 of the published sequence of MCPyV in GenBank (NCBI) (EU375803) [2] and that obtained from the sequencing of rectal swabs positive for MCPyV NCCR (*MCPyV 4,7,8,12,13,14,21,23,26,29,34,35,36,37,38,39,43,55,57,61,64,65,66*). 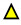
AP-1: 5104–5114 nucleotide position (5′-GATGATGCCCC-3′); 5164–5172 nucleotide position (5′-CTTTGTAAG-3′); 5257–5265 nucleotide position (5′-GTTTATCAG-3′); 5261–5269 nucleotide position (5′-ATCAGTCAA-3′); 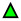
NF1: 5088–5105 nucleotide position (5′-TATTGGCCAGCAGTGTG-3′); 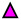
TST1: 5200–5214 nucleotide position (5′-GTTGAAAAAAAGTTA-3′); 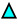
OCT1: 5116–5129 nucleotide position (5′-CATCCTGAAAAATA-3′); 5143–5146 nucleotide position (5′-ACTCTTTTAATG-3′); 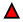
NFκB: 5188–5197 nucleotide position (5′-CGGGCCTCCC-3′); 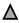
TATA-like sequences: 5146–5160 nucleotide position (5′-CTTTTAATGTCCTCC-3′); 5202–5212 nucleotide position (5′-TGAAAAAAAG-3′); 5256–5265 nucleotide position (5′-TGTTTATCAG-3′); E: experienced HIV-1-positive patients; N: naive HIV-1-positive patients.

The concomitant analysis of 23 NCCR regions amplified from the 23/66 (35%) MCPyV-positive rectal swabs (MCPyV 4,7,8,12,26,29,34,35,37,39,57,61,65,66 belonging to experienced (E) patients and *MCPyV 13,14,21,23,36,38,43,55,64* belonging to naïve (N) patients) showed the presence of several mutations (Figure 4). Deletions were detected throughout the sequence between nucleotides 5094 and 5260 (Figure 4). The 5101 T to G and the 5102 G to T nucleotide transversions were detected in the strains *MCPyV 4, 8, 14, 34, 39*, and *61*, whereas the 5098 C to G nucleotide transversion was found in *MCPyV 35, 36, 38, 43, 55*, and *57*. The 5109 T to C nucleotide transition characterized the strains *MCPyV 4, 8, 12, 21, 34, 39,* and *61* (Figure 4). In *MCPyV 13, 14, 23, 26, 29, 35, 36, 37, 38, 39, 55, 57,* and *66,* the 5148 T to C transition was detected (Figure 4). The 5171, 5175, and 5176 A to T nucleotide transversions, the 5220 T to C transition, and the G to C transversion, located at position 5252 in *MCPyV 34* and *55*, were frequently detected in multiple MCPyV strains, as shown in Figure 4. Lastly, the GTT insertion at positions 5210–5211 in *MCPyV 4, 7, 8, 29, 35, 38, 39*, and *61*, and the GTTGA insertion at positions 5210–5211 in *MCPyV 12, 13, 36, 37, 43*, and *64* were also found (Figure 4). 

Nucleotide substitutions are in bold red text. Deletions are indicated by bold and red dashes. The area highlighted in light gray represents overlapping areas within one or more binding sites. The number of base pair (bp) of each sequence is reported on the right of the Figure.

No significant association was found between the presence of GTT and GTTGA insertions and age and HIV patient’s status (naïve/experienced). It is interestingly to note that the presence of insertions was exclusively found in male gender with a statistical difference very close to significance (*p* = 0.054). 

The main NCCR modifications observed in MCPyV-positive patients, relative to analyzed samples, are summarized in Table 3.

### 3.3. Analysis of Putative Binding Sites in NCCR Sequences

Analysis of putative binding sites was carried out on the NCCR sequence of the prototype MCC350 [2], ranging in nucleotide positions from 5077 to 5280, using TFBIND software, available at http://tfbind.hgc.jp/ [29]. Results showed the presence of putative binding sites for several transcription factors, including NF1, NFκB, Tst-1, OCT1, AP-1, and TATA (Figure 1). Specifically, the NF1 binding site (5′-TATTGGCCAGCAGTGTG-3′) was found into nucleotide positions 5088–5105 whereas the single NFκB (5′-CGGGCCTCCC-3′) was identified into nucleotide positions 5188–5197 (Figure 1). A Tst-1 element 5′-GTTGAAAAAAAGTTA-3′ was observed in 5200–5214 nucleotide positions. Double sequences corresponding to OCT1 binding sites were detected at nucleotide positions 5116–5129 (5′-CATCCTGAAAAATA-3′) and 5143–5146 (5′-ACTCTTTTAATG-3′). Moreover, multiple binding sites for AP-1 were present and placed in the following nucleotide positions: 5104–5114 (5′-GATGATGCCCC-3′), 5164–5172 (5′-CTTTGTAAG-3′), 5257–5265 (5′-GTTTATCAG-3′), and 5261–5269 (5′-ATCAGTCAA-3′) (Figure 1). Additionally, three TATA-like sequences were inserted into nucleotide positions 5146–5160 (5′-CTTTTAATGTCCTCC-3′), 5202–5212 (5′-TGAAAAAAAG-3′), and 5256–5265 (5′-TGTTTATCAG-3′) (Figure 1). Further DNA putative binding motifs, such as Sp1 and/or p53, already found in NCCR of other HPyVs [13,30] were not seen in reference NCCR MCC350 sequence. All sequences, recovered from MCPyV-positive urine, plasma, and rectal swabs, isolated from the enrolled HIV-1-positive population, were compared to NCCR (5077–5280 bp) reference sequence MCC350 [2] for the examination of the cellular transcription binding sites as reported above. Results evidenced that in urine, the putative NF1 binding site (nucleotide positions 5088–5105) was maintained in all analyzed strains, as well as AP-1 in 5164–5172 nucleotide position, NFκB in 5188–5197 nucleotide position, Tst-1 in 5200–5214 nucleotide position, and TATA in 5202–5212 nucleotide position (Figure 2). Between nucleotide positions 5104–5114, 5116–5129, and 5146–5160, the AP-1, OCT1, and TATA elements were altered in several samples, as shown in Figure 2. In plasma, all MCPyV strains displayed several alterations within the putative binding sites allocated between 5077 and 51160 nucleotide positions (Figure 3). As observed in urine, 5164–5172 AP-1 putative binding site was always retained in all sequences. NFκB in 5188–5197 nucleotide positions resulted incomplete in *MCPyV 26* and *35,* whereas in *MCPyV 42,* it was characterized by a G to C transversion (Figure 3). Insertions and deletions altered the sequences of 5200–5214 Tst-1 and 5202–5212 TATA elements in all *MCPyV* samples with the exception of *MCPyV 21* and *41* (Figure 3). Among nucleotide positions 5246–5280, *MCPyV 36* conserved the deletions found in urine, altering the canonical configuration of AP-1 and TATA putative sites (Figure 3). Regarding sequences recovered from MCPyV-positive rectal swabs, although all strains showed an unchanged NFκB element, several alterations, including deletions or single base substitutions, were detected throughout the NCCR sequence (Figure 4). Interestingly, as previously described for plasma, the GTT and GTTGA insertions, into nucleotide positions 5210–5211, changed the sequence of TSt-1 and TATA elements in *MCPyV 4, 7, 8, 12, 13, 14, 29, 35, 36, 37, 38, 39, 43, 64,* and *65* (Figure 4).

## 4. Discussion

A direct link between immunosuppression and the development of opportunistic infections has been established, and it is also known that HPyVs, that commonly infects healthy humans, have a clearly established potential for causing severe organ damage or malignant transformation, especially in individuals with weakened immunity [31,32,33,34]. Although it is well defined that HIV/AIDS predisposes to viral infection and to development of MCC, up to now, very few studies focused on MCPyV prevalence and viral load in HIV-1-positive individuals without MCC [35]. Published data demonstrated that levels of anti-MCPyV IgG in HIV/AIDS patients were significantly higher than those in non-AIDS HIV-infected patients and the prevalence of MCPyV-DNA in peripheral blood mononuclear cells (PBMCs) of HIV/AIDS and non-AIDS HIV-infected patients were 17% and 16%, respectively [36]. Moreover, Fukumoto and colleagues found that 9/23 (39%) serum samples from HIV patients, without highly active antiretroviral therapy (HAART) therapy, were MCPyV-positive [37]. Lastly, MCPyV DNA was found in 2/19 (11%) urine samples of HIV patients, as reported by Torres and colleagues [38].

In this framework, the aim of our study was to evaluate the prevalence and MCPyV loads in urine, plasma, and rectal swabs, among an HIV-1-positive population, in order to better understand MCPyV tissue tropism and to provide new insights on the possible pathogenic role of this virus in human diseases. Our results showed that MCPyV DNA was detected in all type of analyzed specimens, suggesting that the renal epithelium, blood, and gastrointestinal tract could be considered a target of the virus infection. The viral DNA detection confirms that MCPyV is widespread among the population, supporting the urine–oral and fecal–oral routes of transmission [39,40]. In our patients, higher MCPyV prevalence and a higher amount of MCPyV DNA were found in rectal swabs compared with urine and plasma samples. Since some authors reported evidence of HPyVs infection in anal/rectal samples from men who had sex with men, both in HIV-1-positive and in negative population, with higher frequency of MCPyV infection, it is possible to suggest also the sexual route as a possible route of transmission of HPyVs in humans [5,40,41].

Moreover, as suggested by Vergori and colleagues, the higher MCPyV prevalence in rectal swabs could be related to differences in local mucosal immunity activity. In other sites of infection, viral reactivation could be less likely when compared with anal/rectal compartments, where traumatisms and the occurrence of sexually transmitted diseases may decrease the ability of the host to clear virus [5,42]. It is also plausible that the renal epithelium and blood may represent latent sites of the virus, rather than a site of active replication. This assumption is in agreement with HPyVs biology: it is well recognized that primary HPyVs infection is followed by the establishment of an asymptomatic latency state, possibly in the lymphoid, neuronal, kidney, hematopoietic tissues, characterized by low-level replication and excretion, for example, in urine [10,43]. Although no significant association was found between the presence of MCPyV DNA and HIV patient’s status at enrollment, MCPyV DNA was found in a higher percentage in naïve patients compared with experienced ones, either in single or all anatomical sites. This data suggest that a weakened immune system with low CD4+ counts as often reported in naïve patients might favor MCPyV replication. In order to improve the knowledge of NCCR alterations in MCPyV strains circulating in the context of immunosuppression, the NCCR variability was analyzed. NCCR rearrangements are described as a pivotal event in the onset of HPyVs-related pathology, as demonstrated for JCPyV and BKPyV in which NCCRs not only control gene expression but also serve as main determinants in viral replication, containing the origin of DNA replication and transcription factor binding sites [15,44]. Instead, little is known regarding the role of NCCR in MCPyV infection, and limited data are available about the relationship between MCPyV NCCR strains and MCPyV pathogenesis. NCCR sequence analysis revealed a high degree of homology with MCC350 strain in urine, whereas transitions, transversions, and single or double deletions were observed in plasma and rectal swabs. Differently to JCPyV and BKPyV, in which the early proximal side of NCCR is highly conserved and the late proximal side undergoes rearrangements [15], we found that insertions and deletions occurred both in early and in late proximal side of the MCPyV NCCR. Specifically, in the analyzed strains, representative GTTGA and GTT insertions (nucleotide positions 5210–5211) were observed in both plasma and rectal swabs. Interestingly, these alterations were found exclusively in male gender with a statistical difference very close to significance (*p* = 0.054). The MCPyV NCCR structure, in contrast to JCPyV and BKPyV, has been previously associated with the geographic origin of the patients [13]. Hashida and colleagues identified two major subtypes of MCPyV NCCR, subtypes I and II, with the presence or absence of a 25 bp tandem repeat (TGTCCTCCTCCCTTTGTAAGAGAAA) into nucleotide positions 5177–5178, respectively. Based on the occurrences of two additional insertions (2 bp, TT, and 5 bp insertions, GTTGA, between nucleotide positions 5199–5200 and 5210–5211, respectively), MCPyV strains were assigned further to five genotypes. In our analyzed strains, we found the MCPyV NCCR IIa-2 strain in plasma and rectal swabs, which contain the 5 bp insertion and represents the predominant strain among white persons of European descent, as predictable for our cohort of patients [13]. In this study, we evaluated beside the NCCR rearrangements the possible nucleotide changes that fell within the putative binding sites for cellular transcription factors [13,30,44,45]. Sequence analysis showed that MCC350 NCCR sequence contains the NF1, NFκB, Tst-1, OCT1, AP-1, and TATA binding sites already described within the NCCRs of other HPyVs [13,30,44,45]. In several strains obtained from MCPyV-positive samples, deletions, insertions, or single base substitutions fell within these putative binding sites. It can be envisaged that some of these changes did not allow the identification of putative binding motifs such as Sp1 and/or p53, already described in the NCCR of other HPyVs [13], in our NCCR sequences. The relevance of cellular factors such as Tst-1, NF-1, Sp1, NF-kB, and PURα, that specifically determine JCPyV tropism for glial cells and play an important role in efficient HPyVs DNA replication, is well described [46]. Moreover, a potential association, between a C/G mutation in the NCCR Sp1 site and increased BKPyV virulence in hemorrhagic cystitis (HC) patients, has been proposed [47]. In light of these findings, further studies are warranted in order to define the importance of these NCCR binding sites and understand how their changes (mutations, insertions, or deletions) may drive MCPyV replication and subsequent in vivo pathogenicity. Finally, in the attempt to understand whether rearrangements could be correlated to a higher replicative capacity, the median MCPyV viral load of each district was analyzed in relation to the presence of *rr*-NCCRs, confirming that the MCPyV strains that carried the GTTGA insertion in NCCR showed a higher rate of replication compared with the ones without insertion, either in the same or different anatomical sites. As previously reported for BKPyV and JCPyV, *rr*-NCCRs conferred a higher replication rate to these viruses contributing to diseases progression [22]. Consequently, also for MCPyV, it is possible to speculate that the shift from canonical NCCR to *rr*-NCCR could determine higher replication capacity and increase the pathogenic potential in the context of immunosuppression. It is also likely that high MCPyV-DNA levels might increase the chance of its integration into the host cell genome and, then, its oncogenic properties in a context different from that of MCC.

## 5. Conclusions

Based on the high prevalence of MCPyV in rectal swabs, we suggest that sexual route can represent another route of transmission of HPyVs among humans. Although sequencing analysis revealed the presence of numerous mutations in the NCCR, whether these mutations may have an impact on the pathogenic features of the virus remains to be determined. The study adds important information about MCPyV prevalence, viral load, and NCCR behavior in HIV-positive individuals and suggests to monitor the possible role of MCPyV in triggering disease outside the MCC context.

## Figures and Tables

**Table 1:** Demographics and clinical features of HIV-1-positive patients at enrollment.

Patients Enrolled	Total *n*.	Gender, *n*.	Age	HIV-1 RNA load, Range	CD4+ Counts, Range
**Naive**	22	Male: 16; Female: 6	Range: 21–68 years old Mean: 39.27 years old	1.26 × 10^3^–10 × 10^6^ copies/mL	9–890/mm^3^
**Experienced**	44	Male: 39; Female: 5	Range: 21–76 years old Mean: 43.7 years old	TND-27 × 10^5^ copies/mL	312–1178/mm^3^

*n*: number; TND: Target not detected.

**Table 2:** Detection and quantification of Merkel cell polyomavirus (MCPyV) DNA by real-time qPCR.

HIV-1 Status Patients, n.	MCPyV+ Patients, n.	MCPyV+ Samples		
**Naive**	**Naive**	**Urine**	**Plasma**	**Rectal Swab**
22/66	12/22	5	4	9
**Experienced**	**Experienced**			
44/66	19/44	5	3	14
**HIV-1 Status**	**MCPyV DNA Detection %**			
	**Overall Sites**	**Urine**	**Plasma**	**Rectal Swab**
**Naive**	54.55	22.73	18.18	40.91
**Experienced**	43.18	11.36	6.82	31.82
		**qPCR results (median value)**		
	**Urine**	**Plasma**	**Rectal Swab**	
	5.15 × 10^2^ copies/mL (IQR 4.5 × 10^2^–5.4 × 10^2^)	1.5 × 10^2^ copies/mL (IQR1.3 × 10^2^–2.0 × 10^2^)	2.3 × 10^3^ copies/mL (IQR1.8 × 10^3^–4.1 × 10^3^)	

n: number; MCPyV+: MCPyV DNA positive.

**Table 3:** Summary of the main NCCR modifications observed in MCPyV-positive patients relative to analyzed samples.

Patients, n°	N/E	Age	GENDER	NCCR MODIFICATIONS		
				URINE	PLASMA	RECTAL SWAB
4	E	29	M			∆GCA, ∆CC, insGTT
7	E	36	M			∆GCA, ∆CCC, insGTT
8	E	46	M			∆AA, insGTT
11	E	55	M	∆CC, ∆AA		Not detected
12	E	37	M			∆CC, ∆TT, insGTTGA
13	N	34	M		∆CC, ∆AA, insGTTGA	∆CCC, insGTTGA
14	N	68	M			∆GCA, ∆AA, ∆GA, insGTTGA
19	N	42	M	∆CC		Not detected
21	N	36	M		∆CC, ∆AA	∆AA, ∆TTG
23	N	50	M			∆ATG, ∆CC, ∆AA
26	E	41	F		∆CC, ∆AA	∆TGA, ∆ATG, ∆CC, ∆AA
27	E	38	M	∆CC, ∆AA		
29	E	38	M			∆GA, ∆CC, ∆AA, ins GTT
30	E	39	F	∆CCCCCC		Not detected
34	E	30	M			∆CC, ∆AA
35	E	40	M		∆CC	∆CC, insGTT
36	N	41	M	∆AA, ∆TTTGTT	∆AA, ∆TTTTGTTT, insGTTGA	∆GA, ∆ATG, 2∆AA, ∆TTTTGTTT, insGTTGA
37	E	61	M			∆GA, ∆CC, ∆AA, insGTTGA
38	N	45	M	∆AA		∆CCCC, 2∆AA, insGTT
39	E	25	M			∆GCA, ∆CCC, insGTT
40	E	26	M	Not detected		
41	N	28	M		∆AA	Not detected
42	E	28	M		Not detected	Not detected
43	N	46	M			insGTTGA
47	N	44	M	Not detected		
55	N	45	M			∆ATG, ∆CC
57	E	34	M			∆TGATGA, ∆CC, ∆AA
61	E	43	M			∆GCA, ∆CCAA, insGTT
64	N	45	F	Not detected		∆GA, ∆CC, ∆CT, insGTTGA
65	E	60	M	Not detected		∆GA, ∆ATG, ∆CC, ∆AAAAA, insGTTGA
66	E	44	F			∆GA, ATG, ∆CC, ∆AAAAA

n°: number of patients; N: naïve: E: experienced; ins: insertion; ∆: nucleotide deletion.

## References

[1] Barth H., Solis M., Kack-Kack W., Soulier E., Velay A., Fafi-Kremer S. (2016). In Vitro and in Vivo models for the study of Human Polyomavirus infection. Viruses.

[2] Feng H., Shuda M., Chang Y., Moore P.S. (2008). Clonal integration of a polyomavirus in human Merkel cell carcinoma. Science.

[3] Tolstov Y.L., Pastrana D.V., Feng H., Becker J.C., Jenkins F.J., Moschos S., Chang Y., Buck C.B., Moore P.S. (2009). Human Merkel cell polyomavirus infection II: MCV is a common human infection that can be detected by conformational capsid epitope immunoassays. Int. J. Cancer.

[4] Prezioso C., Di Lella F.M., Rodio D.M., Bitossi C., Trancassini M., Mele A., de Vito C., Antonelli G., Pietropaolo V. (2019). Merkel cell Polyomavirus DNA detection in respiratory samples: Study of a cohort of patients affected by cystic fibrosis. Viruses.

[5] Zanotta N., Delbue S., Signorini L., Villani S., D’Alessandro S., Campisciano G., Colli C., De Seta F., Ferrante P., Comar M. (2019). Merkel Cell Polyomavirus Is Associated with Anal Infections in Men Who Have Sex with Men. Microorganisms.

[6] Babakir-Mina M., Ciccozzi M., Lo Presti A., Greco F., Perno C.F., Ciotti M. (2010). Identification of Merkel Cell Polyomavirus in the lower respiratory tract of Italian patients. J. Med. Virol..

[7] Iaria M., Caccuri F., Apostoli P., Giagulli C., Pelucchi F., Padoan R.F., Caruso A., Fiorentini S. (2015). Detection of KI WU and Merkel cell polyomavirus in respiratory tract of cystic fibrosis patients. Clin. Microbiol. Infect..

[8] Prezioso C., Ciotti M., Obregon F., Ambroselli D., Rodio D.M., Cudillo L., Gaziev J., Mele A., Nardi A., Favalli C. (2019). Polyomaviruses shedding in stool of patients with hematological disorders: Detection analysis and study of the non-coding control region’s genetic variability. Med. Microbiol. Immunol..

[9] Schowalter R.M., Buck C.B. (2013). The Merkel cell polyomavirus minor capsid protein. PLoS Pathog..

[10] Delbue S., Franciotta D., Giannella S., Dolci M., Signorini L., Ticozzi R., D’Alessandro S., Campisciano G., Comar M., Ferrante P. (2019). Human Polyomaviruses in the cerebrospinal fluid of neurological patients. Microorganisms.

[11] Ciotti M., Prezioso C., Pietropaolo V. (2019). An overview on human polyomaviruses biology and related diseases. Future Virol..

[12] An P., Sa’enz Robles M.T., Pipas J.M. (2012). Large T antigens of polyomaviruses: Amazing molecular machines. Annu. Rev. Microbiol..

[13] Hashida Y., Higuchi T., Matsui K., Shibata Y., Nakajima K., Sano S., Daibata M. (2018). Genetic variability of the noncoding control region of cutaneous Merkel cell polyomavirus: Identification of geographically related genotypes. J. Infect. Dis..

[14] Moens U., Johansen T., Johnsen J.I., Seternes O.M., Traavik T. (1995). Noncoding control region of naturally occurring BK virus variants: Sequence comparison and functional analysis. Virus Genes.

[15] White M.K., Safak M., Khalili K. (2009). Regulation of gene expression in primate polyomaviruses. J. Virol..

[16] Helle F., Brochot E., Handala L., Martin E., Castelain S., Francois C., Duverlie G. (2017). Biology of the BKPyV: An update. Viruses.

[17] Pietropaolo V., Prezioso C., Bagnato F., Antonelli G. (2018). John Cunningham virus: An overview on biology and disease of the etiological agent of the progressive multifocal leukoencephalopathy. New Microbiol..

[18] Rubinstein R., Schoonakker B.C., Harley E.H. (1991). Recurring theme of changes in the transcriptional control region of BK virus during adaptation to cell culture. J. Virol..

[19] Johnsen J.I., Seternes O.M., Johansen T., Moens U., Mantyjarvi R., Traavik T. (1995). Subpopulations of non-coding control region variants within a cell culture-passaged stock of BK virus: Sequence comparisons and biological characteristics. J. Gen. Virol..

[20] Prezioso C., Scribano D., Bellizzi A., Anzivino E., Rodio D.M., Trancassini M., Palamara A.T., Pietropaolo V. (2017). Efficient propagation of archetype JC polyomavirus in COS-7 cells: Evaluation of rearrangements within the NCCR structural organization after transfection. Arch. Virol..

[21] Prezioso C., Scribano D., Rodio D.M., Ambrosi C., Trancassini M., Palamara A.T., Pietropaolo V. (2018). COS-7-based model: Methodological approach to study John Cunningham virus replication cycle. Virol. J..

[22] Gosert R., Rinaldo C.H., Funk G.A., Egli A., Ramos E., Drachenberg C.B., Hirsch H.H. (2008). Polyomavirus BK with rearranged noncoding control region emerge in vivo in renal transplant patients and increase viral replication and cytopathology. J. Exp. Med..

[23] Olsen G.H., Hirsch H.H., Rinaldo C.H. (2009). Functional analysis of polyomavirus BK non-coding control region quasispecies from kidney transplant recipients. J. Med. Virol..

[24] Gosert R., Kardas P., Major E.O., Hirsch H.H. (2010). Rearranged JC virus noncoding control regions found in progressive multifocal leukoencephalopathy patient samples increase virus early gene expression and replication rate. J. Virol..

[25] Saiki R.K., Bugawan T.L., Horn G.T., Mullis K.B., Erlich H.A. (1986). Analysis of enzymatically amplified beta-globin and HLA-DQ alpha DNA with allele-specific oligonucleotide probes. Nature.

[26] Imajoh M., Hashida Y., Nemoto Y., Oguri H., Maeda N., Furihata M., Fukaya T., Daibata M. (2012). Detection of Merkel cell polyomavirus in cervical squamous cell carcinomas and adenocarcinomas from Japanese patients. Virol. J..

[27] Hashida Y., Imajoh M., Nemoto Y., Kamioka M., Taniguchi A., Taguchi T., Kume M., Orihashi K., Daibata M. (2013). Detection of Merkel cell polyomavirus with a tumour-specific signature in non-small cell lung cancer. Br. J. Cancer.

[28] ClustalW2-Multiple Sequence Alignment. http://www.ebi.ac.uk/clustalw/.

[29] TFBIND. http://tfbind.hgc.jp/.

[30] Raj G.V., Khalili K. (1995). Transcriptional regulation: Lessons from the human neurotropic polyomavirus, JCV. Virology.

[31] Engels E.A., Frisch M., Goedert J.J., Biggar R.J., Miller R.W. (2002). Merkel cell carcinoma and HIV infection. Lancet.

[32] Clarke C.A., Robbins H.A., Tatalovich Z., Lynch C.F., Pawlish K.S., Finch J.L., Hernandez B.Y., Fraumeni J.F., Madeleine M.M., Engels E.A. (2015). Risk of Merkel cell carcinoma after solid organ transplantation. J. Natl. Cancer Inst..

[33] Rotondo J.C., Bononi I., Puozzo A., Govoni M., Foschi V., Lanza G., Gafà R., Gaboriaud P., Touzé F.A., Selvatici R. (2017). Merkel cell carcinomas arising in autoimmune disease affected patients treated with biologic drugs, including anti-TNF. Clin. Cancer Res..

[34] McIlroy D., Halary F., Bressollette-Bodin C. (2019). Intra-patient viral evolution in polyomavirus-related diseases. Phil. Trans. R. Soc. B.

[35] Goldstein R.H., DeCaprio J.A. (2019). Merkel Cell Carcinoma in the HIV-1/AIDS Patient. Cancer Treat Res..

[36] Vahabpour R., Nasimi M., Naderi N., Salehi-Vaziri M., Mohajel N., Sadeghi F., Keyvani H., Monavari S.H. (2017). Merkel cell polyomavirus IgG antibody levels are associated with progression to AIDS among HIV-infected individuals. Arch. Virol..

[37] Fukumoto H., Sato Y., Hasegawa H., Katano H. (2013). Frequent detection of Merkel cell polyomavirus DNA in sera of HIV-1-positive patients. Virol. J..

[38] Torres C., Barrios M.E., Cammarata R.V., Cisterna D.M., Estrada T., Martini Novas S., Cahn P., Blanco Fernández M.D., Mbayed V.A. (2016). High diversity of human polyomaviruses in environmental and clinical samples in Argentina: Detection of JC, BK, Merkel-cell, Malawi, and human 6 and 7 polyomaviruses. Sci. Total Environ..

[39] Wieland U., Mauch C., Kreuter A., Krieg T., Pfister H. (2009). Merkel cell polyomavirus DNA in persons without Merkel cell carcinoma. Emerg. Infect. Dis..

[40] Moens U., Krumbholz A., Ehlers B., Zell R., Johne R., Calvignac-Spencer S., Lauber C. (2017). Biology, evolution, and medical importance of polyomaviruses: An update. Infect. Genet. Evol..

[41] Peng J., Li K., Zhang C., Gao L., Jin Q. (2016). Human papillomavirus and polyomavirus coinfections among Chinese men who have sex with men. J. Infect..

[42] Vergori A., Garbuglia A.R., Piselli P., Del Nonno F., Sias C., Lupi F., Lapa D., Baiocchini A., Cimaglia C., Gentile M. (2018). Oral human Papillomavirus DNA detection in HIV-positive men: Prevalence, predictors, and co-occurrence at anal site. BMC Infect. Dis..

[43] Signorini L., Belingheri M., Ambrogi F., Pagani E., Binda S., Ticozzi R., Ferraresso M., Ghio L., Giacon B., Ferrante P. (2014). High frequency of Merkel cell polyomavirus DNA in the urine of kidney transplant recipients and healthy controls. J. Clin. Virol..

[44] Ajuh E.T., Wu Z., Kraus E., Weissbach F.H., Bethge T., Gosert R., Fischer N., Hirsch H.H. (2018). Novel human Polyomavirus noncoding control regions differ in bidirectional gene expression according to host cell, Large T-antigen expression, and clinically occurring rearrangements. J. Virol..

[45] Markowitz R.B., Tolbert S., Dynan W.S. (1990). Promoter evolution in BK virus: Functional elements are created at sequence junctions. J. Virol..

[46] Safak M., Gallia G.L., Khalili K. (1999). A 23-bp sequence element from human neurotropic JC virus is responsive to NF-kappa B subunits. Virology.

[47] Priftakis P., Bogdanovic G., Kokhaei P., Mellstedt H., Dalianis T. (2003). BK virus (BKV) quantification in urine samples of bone marrow transplanted patients is helpful for diagnosis of hemorrhagic cystitis, although wide individual variations exist. J. Clin. Virol..

